# A qualitative study of cancer survivors’ exercise behaviour 4 months after a telerehabilitation program

**DOI:** 10.1007/s00520-026-11016-y

**Published:** 2026-09-08

**Authors:** Amy M. Dennett, Casey L. Peiris, Made U. Rimayanti, Christian Barton, Nicholas F. Taylor, Katherine E. Harding, Germaine Tan, Allison Ezzat, Nora Shields

**Affiliations:** 1 School of Allied Health, Human Services and Sport, La Trobe University, Melbourne, VIC Australia; 2 Allied Health Clinical Research Office, Eastern Health, Melbourne, VIC Australia; 3 Allied Health Department, The Royal Melbourne Hospital, Parkville, Australia; 4 Department of Physical Therapy, University of British Columbia, Vancouver, Canada; 5 School of Psychology and Public Health, Olga Tennison Autism Research Centre, La Trobe University, Melbourne, VIC Australia

**Keywords:** Telehealth, Telerehabilitation, Cancer, Exercise, Behavior change

## Abstract

**Purpose:**

Although telerehabilitation can improve access to exercise programs for cancer survivors, it is not known if these programs lead to ongoing change in exercise habits. This study explored participant experiences of exercise 4 months after finishing specialized cancer exercise-based telerehabilitation.

**Method:**

A qualitative study embedded in a randomized controlled trial evaluated exercise-based cancer telerehabilitation delivered in groups. Data were collected via semistructured interviews that were audio-recorded and transcribed verbatim. Seventeen adult cancer survivors (age 21 to 80) were purposively sampled 4 months after completing exercise-based cancer telerehabilitation. Data were coded independently by two researchers and analyzed inductively within an interpretive description framework.

**Results:**

The overarching theme was telerehabilitation was perceived to facilitate positive exercise intentions. Participants said they were empowered to exercise through knowledge, opportunity, and connection gained through telerehabilitation. They described acting on their positive intentions to exercise to varying degrees following telerehabilitation, depending on their context. A subtheme was that exercise was challenging in their new reality created by cancer. Comorbidities, ongoing side effects, previous exercise experience, and personal factors were considered by some to influence their ability to exercise.

**Conclusion:**

Telerehabilitation may facilitate positive intent to maintain exercise. Cancer survivors may need ongoing support after telerehabilitation to act on positive exercise intentions due to health-related difficulties.

**Implications for cancer survivors:**

Participation in telerehabilitation may be a positive first step to initiate exercise, but ongoing support to maintain positive behavior changes is likely to be needed for people without previous exercise experience.

**Supplementary information:**

The online version contains supplementary material available at https://doi.org/10.1007/s00520-026-11016-y.

## Introduction

Exercise-based rehabilitation is recommended as part of best practice cancer care [1, 2]. Participation in regular exercise can mitigate adverse treatment effects, improve physical and psychological function, and enhance quality of life [2, 3]. Exercising long term has also been found to improve disease-free survival by 28% and overall survival by 37% [4]. As cancer is increasingly recognized as a chronic disease, physical activity including regular exercise is important for optimizing long-term health. Cancer treatment can be a teachable moment for changing behavior [5], but few suitable cancer exercise services exist worldwide that are delivered and supervised by staff trained in exercise oncology to facilitate and maintain exercise and physical activity [6].

Participating in exercise can be difficult after a cancer diagnosis. Cancer survivors entering rehabilitation typically have low levels of physical activity, on average completing just 12 min per day of moderate to vigorous activity [7] and half report exercising less than 2 days per week [8]. Many factors contribute to this including experiencing debilitating treatment side effects such as fatigue [9] and juggling competing medical demands [10]. Convenience of exercise programs, social support, and access to expert instructors are reported as key facilitators to participation [10]. Supervised, exercise-based rehabilitation programs may provide a positive first step to changing exercise behaviors. Exercise‑based cancer rehabilitation is the targeted prescription of exercise delivered by health professionals, as a therapeutic intervention to prevent, mitigate, or reverse cancer and treatment‑related impairments, thereby improving function, symptom burden, and quality of life in people with cancer [11, 12]. A recent review including nine trials found a small effect for improving physical activity levels 6 months after completing *in-person* exercise-based rehabilitation after cancer [3]. However, many cancer survivors report difficulty with being physically active in the form of exercise once structured rehabilitation has finished [13–15].

Telerehabilitation may overcome barriers to participating in exercise with in-person supervision. Technologies such as videoconferencing, wearable devices, and mobile applications enable participants to exercise in their own environment [16]. These tools help maintain key elements of in-person rehabilitation such as exercise demonstration, instruction, monitoring, and expert guidance. Group-based exercise cancer telerehabilitation is safe and feasible [17, 18]. However, recent studies suggest participants have difficulty developing social connection when accessing telerehabilitation services, which can be an important motivator to exercise [16, 19].

Given exercise behavior change is complex, understanding how supported group cancer telerehabilitation programs influence exercise maintenance is important. Previous qualitative studies have found the transition to ongoing, independent exercise difficult once participants have been discharged from *in-person* rehabilitation as they valued the accountability, professional guidance, and peer support of exercising in a group [13–15]. Exploring how programs may facilitate maintenance of exercise behavior from the participants’ perspective after telerehabilitation is valuable as the drivers of behavior change may differ between telehealth and in-person programs. For example, social connection may be limited or absent when group exercise is performed via telehealth [16]. Telerehabilitation may have specific contributions to exercise behavior change such as facilitating accountability to exercise through the use of activity trackers, the home environment, and connection with skilled staff [20]. No studies have explored how exercise rehabilitation delivered using telehealth influences the ability to maintain exercise after telerehabilitation is complete. Therefore, understanding the medium-term effect of telerehabilitation from the participant perspective may help to identify strategies to further improve exercise levels of people with cancer and optimize outcomes. Therefore, the aim of this study was to explore cancer survivors’ perspectives of their participation in exercise after completing telerehabilitation and factors influencing exercise engagement 4 months after completing a group-based telerehabilitation program.

## Method

### Research design, theoretical framework, and setting

A qualitative study using interpretive description [21] was conducted. Interpretive description is a flexible approach underpinned by a relativist ontology (recognizing multiple realities of experience) and a constructivist epistemology (knowledge coconstructed between researcher and participant). This approach can help address complex experiential questions while producing practical outcomes [21]. This study was embedded within a clinical trial evaluating the effect of a group exercise-based cancer telerehabilitation program (TeleCaRe) (ANZCTRN12621001417875) compared to a single session of assessment and advice delivered by a physiotherapist [22]. Full results of this trial are reported elsewhere [23]. The trial was completed at a large, publicly funded health service extending from metropolitan Melbourne to regional Victoria, Australia. Semistructured interviews captured detailed accounts of participants. Data were collected between June 2022 and December 2023. The study is reported consistent with the Consolidated Criterion for Reporting Qualitative Research Checklist (COREQ) [24] and was approved by hospital and university ethics committees (E21-012–74698). All participants provided written informed consent.

### Participants

Participants were recruited from the experimental group of the TeleCaRe trial [22]. Key eligibility criteria for the trial were participants who had cancer of any stage or type and were receiving treatment or within 12 months of treatment completion and willing and able to use telehealth. Participants without internet, who were high falls risk, or receiving end of life care were excluded. Participants for the qualitative study were purposively sampled to include people of various ages, genders, cancer types, stages, and adherence to the program 4 months after completing cancer telerehabilitation (6 months from trial commencement). Participants were recruited via telephone and recruitment continued until adequate information power was obtained from the sample based on the quality and characteristics of the data rather than a predetermined sample size [25].

### Intervention

Participants in the trial’s experimental group received an 8-week, twice weekly group exercise telerehabilitation program supervised by a physiotherapist trained in cancer care. The program incorporated information provision, monitoring and feedback through a physical activity device (Fitbit), and access to an online information portal and an individualized home exercise program. All participants, including those in the comparison group, also received an initial in-person consultation with a physiotherapist before commencing telerehabilitation. The program had ongoing recruitment of up to eight participants per group. Telerehabilitation sessions were 60 min in duration with exercises individually tailored to each patient. Exercises included seated or standing aerobics, body-weight exercise (e.g., squats), and using equipment such as resistance bands and free weights. Participants discussed ongoing exercise options and local exercise services one on one with the physiotherapist on program discharge and were offered referral to an appropriate exercise program upon request. The intervention included recognized behavior change techniques including instruction, social support, self-monitoring, feedback, and goal setting [26]. Full details of trial procedures are reported in a published trial protocol [22].

### Data collection

Participants completed interviews via phone or videoconference based on their preference. Interviews were anticipated to be approximately 30 min duration. Interviewers were members of the research team (CB/AE) not involved with the delivery or administration of the telerehabilitation program and were PhD-qualified physiotherapists experienced in qualitative research. A flexible interview schedule ensured relevant topics were covered while allowing participants to discuss their experience in their preferred order (Table 1). The approach also allowed for follow-up questions to obtain further information and adapt the interview schedule as needed.

**Table 1:** Semistructured interview schedule

Topic area	Sample questions
Behavior change	*Tell me about your current exercise levels*
	*How have you applied the information from the cancer telerehabilitation program to change your exercise levels over the past 6 months?*
	*What challenges have you experienced in being able to maintain or change your physical activity over the last 6 months?*
	*What has helped you maintain or change your physical activity over the last 6 months?*
Attributing change	*What motivates you to exercise?(e.g., family support, online resources, written instructions, access to gym/supervision, feeling well)?*
	*How do you think receiving telerehabilitation contributed to your current exercise levels?*
Intention of future change	*What changes, if any would you like to make in the next 6 months to your exercise habits?*
	*What support do you need to make changes to your future exercise habits?*

### Data analysis

Interviews were audio-recorded and transcribed verbatim. Transcripts were deidentified and assigned an identification code for anonymity. Four PhD-qualified researchers (AD/MR/CP/NS) with experience in qualitative research completed data analysis via inductive thematic analysis. The principal researcher (AD) was employed by the health service where the study was conducted and has clinical experience in cancer rehabilitation but was not involved in the delivery of the telerehabilitation intervention. Researchers first read all transcripts to become familiar with the data. Two researchers (AD and MR) then independently conducted open coding in NVivo (QSR International, Version 12), generating initial codes inductively from the data. Preliminary codes were compared and discussed, and a codebook was developed that defined each code and outlined its application. The codebook was iteratively refined throughout the analysis phase where new codes were added or existing codes clarified, merged, or refined through discussion between the coders. Four researchers met regularly to review and agree on the evolving codebook and to resolve discrepancies, ensuring consistency in coding. When codes were agreed upon, researchers collaboratively grouped related codes into broader themes through discussion and consensus. Transcripts were subsequently reread to selectively search for any further data related to the identified themes and ensure no aspects of the themes had been overlooked.

### Trustworthiness

For dependability, member checking was conducted by returning transcripts to participants for feedback to verify transcripts were an accurate reflection of their experiences [27]. Having two researchers (AD, MR) independently code data and four researchers read and reflect on the data enabled a thorough understanding of themes and established credibility and confirmability. Descriptions of participants and the setting enhanced transferability [27].

## Results

Twenty participants were approached for interview and 17 agreed to participate. One participant declined as they were busy, one was unable to be contacted, and another scheduled but did not complete an interview. No new ideas were evident in the final nine transcripts; therefore, no further interviews were conducted [28].

Around half the participants were female (*n* = 9 of 17), and participants were on average 58 years old (range 21 to 80) with 11 receiving chemotherapy during telerehabilitation (Table 2). Thirteen (76%) participants reported one or more comorbidities. Participants attended an average of 10 out of 16 sessions (range 3 to 17 [one person completed an extra session]) of telerehabilitation. Adherence was similar to the overall cohort (average 7 of 16 sessions attended). The final session was completed 4 months prior to the interview. The mean interview duration was 13 min (range 8 to 20 min). During member checking, one participant returned their transcripts with minor grammatical amendments.

**Table 2:** Participant demographics

Gender—female *n (%)*	9 (53)
Age (mean, SD)	58 (19)
Diagnosis *n (%)*	
Breast	8 (47)
Prostate	2 (12)
Hematological malignancy	6 (35)
Colorectal	1 (6)
Disease stage *n (%)*	
Early (0–III)	8 (47)
Advanced (IV)	3 (18)
Unknown	6 (35)
Receiving chemotherapy treatment	11 (65)
Treatment status at interview	
Ongoing	8 (47)
Completed	9 (53)
Time since diagnosis (months)	14 (20)
Comorbidities	
Cardiovascular	9 (53)
Respiratory	3 (18)
Endocrine	3 (18)
Musculoskeletal	7 (41)
None	4 (24)
Total *n* =	17

### Overarching theme: telerehabilitation facilitated positive exercise intentions

**Fig. 1:**
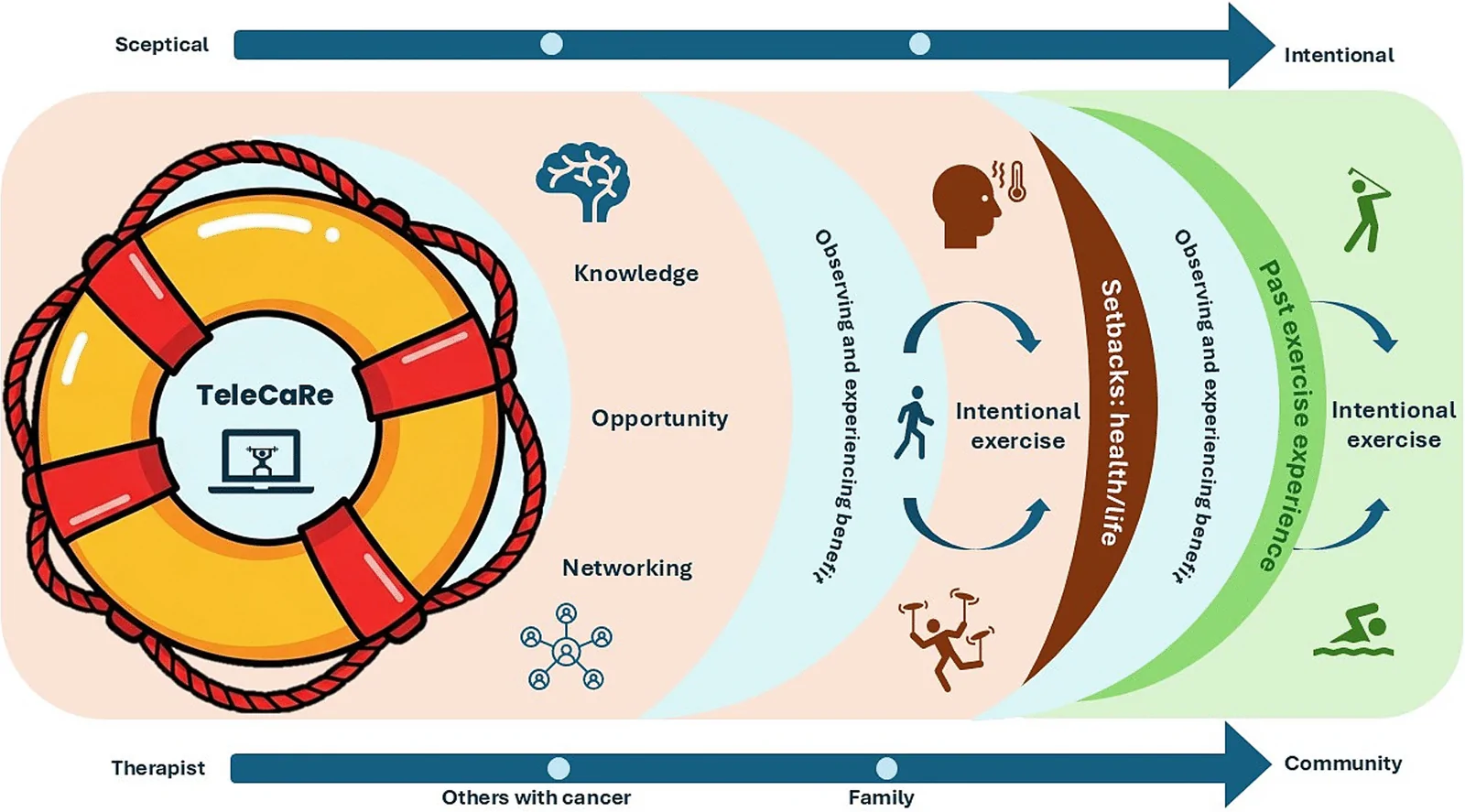
Interpretive synthesis of the effect of the cancer telerehabilitation program facilitating in facilitating a positive cycle of intentional exercise through providing exercise opportunity and its ongoing effect on behavior and networks with others. Green represents the ability to overcome setbacks to routinely participate in exercise. Red represents the context of cancer recovery

The overarching theme was that telerehabilitation provided a lifeline to access exercise rehabilitation and a positive exercise experience that facilitated positive intent to continue to exercise (Fig. 1). A positive downstream effect of acting on exercise intentions with their family and in the community was evident for most participants to varying degrees within the context of setbacks resulting from cancer. This occurred through observing and experiencing the effects of exercise during and after the telerehabilitation program.

The telerehabilitation program was described by participants as a “*lifeline*” that enabled them to improve their health and connect with others after cancer in a convenient and low-cost way. It was said to provide motivation at a time where participants were having difficulties related to cancer. A key component of the program discussed by participants was how telerehabilitation connected them with skilled and empathetic clinicians who were able to impart knowledge about the benefits of exercise and teach them how to exercise safely for their needs within the context of their home.

“*[I was] in that period where it was really, really dark and it just pull[ed] me out of it… I’d lost all my hair. I was feeling awful. I was quite weak…these sorts of things are a lifeline for people like me.*”* (Participant W, breast cancer)*

Participants were able to recall strong reasons for why they should exercise long term after cancer, such as to improve their chance of survival. Participants often reflected on these reasons when detailing their current and future exercise plans.

“*I just find I need to get it done. Because they keep saying that exercise is good for dealing with chemo.*”* (Participant X, colorectal cancer)*

“*I don’t want [cancer] to come back again. And that’s always a concern for us. I think by maintaining and increasing my physical fitness is going to hopefully…keep it away.*”* (Participant W, breast cancer)*

Many participants described how feeling the benefits of exercise during telerehabilitation, such as reducing fatigue and facilitating return to work, prompted them to seek further exercise opportunities when the program ended. Walking with intent was commonly cited as it was convenient. Others had also embedded some of the exercises from the program into their regular routine and/or were exercising in the community by playing golf, lawn bowls, and attending a gym or the swimming pool.

“*it has got me going back to the gym twice a week.*”* (Participant B, multiple myeloma)*

“*I do the exercise from Telecare sometimes, like if I’m tired or the weather’s not good. I do the exercises they show me.*”* (Participant Z, breast cancer)*

Participants said that networking with others during telerehabilitation supported their intentions to continue exercise. In the initial phase of rehabilitation, participants described the benefits of interacting with the therapists and others who had a similar experience in relation to understanding their needs and providing positive feedback. Family was also described as supporting exercise both during and after the program either by participating in exercise with them or by purchasing exercise equipment for the participant. For some, participants spoke about how the program was a conduit to reconnecting with other people in the community.

“*I replace it [the program] mentally in helping out at the Bowls Club with a school’s education programme…up to three times a week…helping small children learn the game of bowls. And that involves exercise…*”* (Participant N, multiple myeloma)*

### Subtheme: exercise was challenging in the new reality created by cancer

The ability of participants to act on their exercise intentions was perceived as challenging in their new reality created by cancer. Comorbidities, ongoing side effects, and contextual factors limited the ability of some participants to exercise after the telerehabilitation program finished despite positive exercise intentions.

“*I was quite sick for quite a period of time…just the pain and feeling really, really unwell was a big challenge for a period…just adjusting to a different life.*”* (Participant W, breast cancer)*

The inability to participate in exercise at the same frequency as during the telerehabilitation program was sometimes expressed as guilt by participants. Some described it being more difficult to exercise independently while managing other factors such as family and work without the structure, accountability, and convenience of being involved in a structured program via telerehabilitation. They often reported a disconnect between knowing they “should” exercise and acting on exercise intentions due to ongoing health problems and changes to their life circumstances.

“*I probably haven’t kept up as much exercise as I should have done partly because of health issues…that’s my fault. That’s not the program’s fault.*”* (Participant B, multiple myeloma)*

Almost all participants discussed how their participation in exercise had been disrupted at some point after completing rehabilitation. Participants described many ongoing health-related issues that impacted their ability to exercise. Common problems included pain and fatigue that were due to side effects of cancer treatment or comorbid conditions. COVID-19 was reported as a barrier to exercise in two ways. Some participants had contracted the disease since finishing telerehabilitation and were physically unable to exercise. Others detailed the desire to return to community gyms or swimming pools but were deterred by the threat of infection due to immunocompromise.

“*I got COVID for a second time. That put things on hold for a little bit.*”* (Participant C, breast cancer)*

However, those with previous exercise experience described being able to overcome obstacles to exercise participation. These participants spoke about the types of exercise they were doing before cancer diagnosis, such as swimming, attending the gym, or simply regular, intentional walking, and how these activities were goals to return to. They also spoke to the benefits of exercise they had previously experienced and enjoyment they had for these activities.

“*I feel like that now that I’ve recovered from cancer, I wanted to get back some of my physical fitness…I used to run a bit so I thought, why not give that a shot?*”* (Participant I, breast cancer)*

Participants who were new to exercise had positive exercise intent but were less likely to act on their intentions. These participants described doing what they could in the form of incidental physical activity around the home or sometimes completing exercises learnt in the program or walking when they were able.

## Discussion

This qualitative study found participation in group exercise-based cancer telerehabilitation facilitated positive exercise intentions which were present 4 months after completing the program. Participants reported experiencing benefits of exercise, which they described as creating a positive cycle of exercise intent, and for some, action, after completing the program. Positive exercise intent persisted to varying degrees even when participants experienced health setbacks. The program was described as a “*lifeline*” because it improved access to exercise. The ability to act on positive intentions varied as it was difficult for some participants to exercise without the structure and support of telerehabilitation or because of a lack of previous exercise experience. Our study confirms previous literature identifying health setbacks as a common barrier to participation in cancer exercise rehabilitation [20, 29, 30] and has suggested how a program can address other perceived barriers, such as lack of exercise experience or lack of structure supporting exercise. It also provides insights on how telerehabilitation might facilitate exercise maintenance behavior from the participants’ perspective, despite published concerns in the literature about health literacy, lack of social support, and inequitable access to technology being potential barriers to telerehabilitation [19, 31].

This study suggests that telerehabilitation can influence positive exercise intent but also highlights the challenges of supporting people to turn this into action after structured programs come to an end. The strongest trial of exercise impacting outcomes of survival and recurrence provided a 3-year exercise intervention with phased levels of support over 3 years to aid exercise maintenance [4]. However, in real-world clinical and community environments, resources are usually insufficient to provide exercise interventions of this duration [6, 32]. Addressing exercise behavior change within structured programs such as telerehabilitation through providing knowledge, capability, and opportunity is consistent with behavior theory [33] as the first step to creating positive exercise behavior change. Ongoing support after telerehabilitation is still needed to maintain action to exercise in the long term, particularly for those who are new to exercise.

The exercise behavior expressed by participants may be explained in part by the transtheoretical model of behavior change [34]. Prior to participating in cancer rehabilitation, whether it be in person or via telehealth as in this study, many people do not feel ready to exercise due to disease and contextual factors acting as a barrier [20]; that is, they are in the precontemplation or contemplation phase of exercise. Partaking in telerehabilitation provides support to transition to the action phase. However, as evidenced in this study and previous literature [13, 14], the transition from action to maintenance or even intent is challenging, particularly when support structures such as therapist supervision are removed.

Recovery after cancer is not linear. Despite completing treatment, some participants experienced persistent challenges to their health that disrupted their ability to exercise moving them back into the contemplation phase, yet they still had positive intent to exercise from the knowledge gained during telerehabilitation. For those with previous exercise experience, and likely high self-efficacy for exercise, this disruption appeared short term. However, for others, transition back to action was described as more difficult. The latter participants may be more likely to lack confidence in their knowledge and body to exercise independently [15].

Flexible models of rehabilitation and community exercise are required to address the varied needs of cancer survivors. Exercise frameworks such as cancer rehabilitation to recreation [35] along with use of triage tools [36] may help guide clinicians to tailor the level of support required as there are important distinctions that can be made between rehabilitation and community-based cancer exercise programs. Exercise-based rehabilitation programs are usually delivered in clinical settings with higher levels of tailoring and oversight by tertiary qualified exercise professionals as opposed to community exercise programs commonly delivered by fitness trainers often with little or no communication with medical teams [35, 37]. For cancer survivors to continue to exercise beyond telerehabilitation, further support in the community is needed to maximize positive exercise intent. For example, all participants described gaining motivation from the knowledge and expert guidance from the physiotherapist. Therefore, cancer survivors would benefit from access to an exercise professional to provide this expert knowledge in the first instance which could be achieved through implementation of physical therapy navigators [38]. Experiencing the benefits of exercise helped sustain the cycle of positive intent during and after the program when cancer survivors were supported by structure, accountability, and social support. Previous exercise experience appeared the strongest determinant of exercise maintenance after telerehabilitation and most exercise completed after the program was conducted without digital support. Given telerehabilitation in clinical settings is usually short term, it is important to be able to identify those with low levels of exercise experience and provide extra support to them in the community whether it be in-person or via telehealth such as through cancer-specific trainer-led programs such as LiveStrong [39] or the Alberta Exercise Program [40] to maintain their exercise levels.

## Strengths and limitations

This study was conducted within a large health service setting using purposive sampling to identify people from a broad age range and cancer experience that enhances generalizability to similar health settings. We believe we achieved adequate information power. Many strategies were employed to ensure rigor and trustworthiness of the data, including independent coding by two reviewers and member checking.

Participants were recruited from a clinical trial and may have been more motivated to exercise than other cancer survivors resulting in selection bias. Adding to this selection bias was the need for participants to have their own device and access to the internet. However, our sample included many participants who were new to exercise, and all participants experienced difficulties with exercise after cancer diagnosis. Just three potential participants in the main trial were ineligible due to lack of device or internet and criteria related to technology. Adherence to the telerehabilitation program was also low indicating potential issues with feasibility of the program. The behavior change elements included in our intervention may have been insufficient to support optimal adherence. However, these features reflect the real-world challenges of delivering telerehabilitation, and we acknowledge experiences of people with higher adherence may be different. The interview duration was relatively short. However, this may reflect the focused aim and themes were consistent among participants and no new ideas were evident in the final nine transcripts, giving us confidence in the data [28]. Data were also limited to participants from a public, metropolitan health service with no cost to participants. Contextual factors such as health literacy, cultural background, and geographic location may influence the experience of people from other areas of the world and their experience exercising after telerehabilitation. Future research may consider investigating the effects of a telerehabilitation program with more explicit behavior change techniques (such as motivational interviewing) on different populations including those from different cultural backgrounds and health systems and those who are highly exercise adherent.

## Conclusion

Telerehabilitation was perceived to facilitate positive intent to exercise through providing opportunity, knowledge, and networking. Ongoing support after telerehabilitation, whether it be in-person or via telehealth, is required for some people to act on their exercise intentions due to ongoing health-related difficulties and setbacks.

## Supplementary information

Below is the link to the electronic supplementary material.

ESM 1 (DOCX 37.8 KB)

## Data Availability

The data generated during and/or analysed during the current study are available in the supplementary file.
