# Supplementary material for: A qualitative study of cancer survivors’ exercise behaviour 4 months after a telerehabilitation program
Source: Support Care Cancer. 2026 Sep 8;34(10):941. doi: 10.1007/s00520-026-11016-y (PMC13553642; doi:10.1007/s00520-026-11016-y)
Supplement: Supplementary file 1 [file 520_2026_11016_MOESM1_ESM.docx]

Supplementary Table 1. Selected quotes

| ***Overarching Theme:* Telerehabilitation facilitated positive exercise intentions** | | | | |
| --- | --- | --- | --- | --- |
| - *“it definitely getting me up getting me moving, really promoted that, you know, not sitting on my, on my bottom feeling sorry for myself, basically, it really got me out of that state. There was a couple of times when I could quite easily not done anything at all.” (Participant W, breast cancer)* | | | | |
| - *“it's just really motivating to, to do an exercise guided by a trainer and also doing exercises together.” (Participant Y, breast cancer)* - *“I want to live, that’s it. If you don't use it, you lose it.” (Participant AC, multiple myeloma)* - *“I’m having these hormone implants every couple of months, and I’ve just piled on about a kilo a month since I’ve started that…that's sort of motivated me a bit more to get into exercising and trying to trim down and get rid of the weight.” (Participant AB, prostate cancer)* - *“walking is gets me to A to B and I've got time on my walk to the shops. Rather than jump in the car” (Participant N, multiple myeloma)* - *“it has got me going back to the gym twice a week.” (Participant B, multiple myeloma)* - *“it'd be nice to see if I can get back to doing that [swimming] plus restarting the exercise zoom meetings would be great again!” (Participant AC, multiple myeloma)* - *“I'm running a little bit more. Although the running program also has a strength and a mobility session” (Participant I, breast cancer)* - *“I'm going to start doing a bit more bike riding again…I'm feeling much better about myself and starting to do some more movement” (Participant AB, prostate cancer)* | | | | |
| **Codes** | | | | |
| **Access/ ‘*lifeline*’** | | | - *“My private health insurance has run out…So these sorts of things are a lifeline for people like me.” Participant W, Breast cancer* - *“you don't expect everything to be on your doorstep for like 20 to 30 minutes…[but other programs] That just adds an hour on to the hour session... And that's just exhausting to me at the thought of it. Which, again, is why the online stuff has just become much more appealing” (Participant V, breast cancer)* - *“I thought it was really helpful, because it got me in touch with a group” (Participant Y, acute myeloid leukemia)* | |
| **Knowledge** | | - *“those few routines that we did during the course that that helped me so to know what to do. So, things I probably knew in the past, but sort of forgotten how to do the exercise” (Participant G, breast cancer)* - *“I have used the resources from [a different rehabilitation program] and the Zoom calls, the exercises, the scheduling and also the sheets.” (Participant N, multiple myeloma)* - *“another concern as well…is I don't want [cancer] to come back again. And that's always a concern for us. I think by maintaining and increasing my physical fitness is going to hopefully…keep it away.” (Participant W, breast cancer)* - *“I just find need to get it done. Because they keep saying that exercise is good for dealing with chemo.” (Participant X, colorectal cancer)* - *“I know that exercise helps me recover faster. And I'd like to recover faster, because my endurance is still not near where I was when I was healthy… it [also] helps my immune system recover as well.” (Participant Y, acute myeloid leukemia)* | | |
| **Seeing and feeling the benefits** | - *“I can see that I'm improving. I could feel like I'm improving. feel like I'm less fatigued. I feel like I have endurance... So the improvements are pretty noticeable.” (Participant Y, Breast Cancer)* - *“the reward for exercising is just that feeling physically well and good. And even bouncing back from having such a horrible experience of getting cancer.” (Participant C, breast cancer)* - *“I'm starting to find that if once I've done the exercise, I feel a lot better. So that keeps me going” (Participant AB, Prostate Cancer)* - *“my cardiologist asked me to do a stress test, we've got stairs here. So I have to do our stairs - 18 up and 18 down so I do anything from 25 to 30 laps…I record my blood pressure and other stats before I undertake it and record them again after and report it. Funnily enough, it’s showing signs of improvement in the sense that I tend to be recovering better and quicker.” (Participant K, Amyloidosis)* - *“I join a walking group every Monday evening for an hour. I do that religiously... because that makes me feel good” (Participant N, Multiple Myeloma)* | | | |
| **Networking** | - *“the physio, ladies were beautiful. And just the other company…comments from the other participants was really good…it was interesting, you know, to see them and sort of the what they were going through as well.” (Participant L, breast cancer)* - *“I know it's good for me [community exercise group] but it also gets me out of the house. And it’s a very social group when we meet up and that’s very pleasant.” (Participant B, multiple myeloma)* - *“I just had a good relationship with the people that I dealt with, and then they understood” (Participant AC, multiple myeloma)* - *“My husband, will look after the kids so I can go for a run or whatever… I did the YWCA [community exercise] program. I found that was really good…because there was women there who had been through similar things to me” (Participant I, breast cancer)* - *“I replace it mentally in helping out at the Bowls Club with a school's education program. So up to three times a week I'm attending the Bowls Club, and helping small children learn the game of bowls. And that involves exercise you're walking is stepping depending down you're picking up heavy balls.” (Participant N, multiple myeloma)* - *“My daughter …she is very good at exercise…she is a complete influence on me. And there she in fact, got me a set of dumbbells for Christmas. … For a while my young granddaughter did the exercises with me. That was really good!” (Participant N, multiple myeloma)* - *“my husband bought me an exercise bike, and some weights, … to encourage me to do some stuff at home when I feel ready to do that. So there's a few options” (Participant P, breast cancer)* - *“I'd see my neighbors. So you know, going, going out and being active was a way of connecting with my friends in the community around me, so. And that, you know, made me feel a bit more normal as well” (Participant W, Breast Cancer)* - *“I was then linked up to other services. So just the networking” (Participant W, breast cancer)* | | | |
| **Initial scepticism** | - *“I was sceptical about whether or not what I would get out of that [telerehabilitation] when I saw our little exercise group. We have various ages.. And I just thought, Okay, well, this will be fun, because, like, it's just nice to connect with people. But it was so good!” (Participant C, breast cancer)* - *“when I got on the Zoom trial it was slightly different, at first I thought, ‘this is not the same. This is just flexibility.’ …But the more I [did] …, wow, it was good. So I my mind was changed.” (Participant N, multiple myeloma)* - *“I'm not big on doing online, stuff like that, if I find it hard to engage, but I actually enjoyed it...it was super practical and productive.” (Participant V, breast cancer)* | | | |
| ***Subtheme: Managing exercise in a new reality*** | | | | |
| - *“At the moment, it's not been going great. I've just started exercising again last week or two. I've had a few issues over the last couple of months… So I'm starting to get back into exercising again. (Participant AB, prostate cancer)* | | | | |
| - *“I was quite sick for quite a period of time and I had to go and have quite a few one for blood transfusions…just the pain and feeling really, really unwell was a big challenge for, for a period…just adjusting to a different life.” (Participant W, breast cancer)* | | | | |
| - *“mid February, I had my surgery…And that took me out for about four weeks. So, I've only just gotten back into being fit” (Participant W, breast cancer)* | | | | |
| **Co-morbidities and side-effects** | - *“my husband I both had COVID that sort of meant that I was very tired and slept a bit I do get very tired and tend to fall asleep quite a lot…I haven't been able to go for many walks this week” (Participant B, multiple myeloma)* - *“I got COVID for a second time. That put things on hold for a little bit” (Participant C, breast cancer)* - *“I just feel that I've got a lot of pain and one of my joints seem to be stiff and painful quite often and I need a little bit of loosening up. So, this is where I thought the aerobic type exercises might just help that along a bit.” (Participant AB, prostate cancer)* - *“I’m toying with the idea of getting back to swimming because I enjoyed that. Problem is the I’d want to go to…the two places that have been a member of [but] getting to and from the water, the place is brewing with kids and it’s too high risk. I’m still at the stage that I do all the preventative things… I’m still wearing a mask.” (Participant K, amyloidosis)* - *“basically, since I finished that initial chemo trial, the new chemo and regime that I'm on has really taken a toll on me…[but] I'm hopeful that we can start [exercising] again ” (Participant AC, multiple myeloma)* - *“because the reason is that I feel petrified health wise by because my body is so weak, after being through a year of chemo treatment...on the top of that, at the end of the year, having all the extra things so at the moment, I would say a very delicate state at this point.” (Participant G, breast cancer)* - *“I've had nerve damage to the feet. So walking and exercising has been very difficult. So I use a walker now when I go out anywhere” (Participant L, breast cancer)* - *“I've done a bone marrow transplant in between these two times. So I'm recovering from that too” (Participant Y, acute myeloid leukemia)* | | | |
| **Life challenges** | - *“my brother…passed away in early December. He had a brain tumor. So that sort of knocked me around a little bit and I didn't become lazy but I think I became a little bit complacent about doing other things in life.” (Participant AB, prostate cancer)* - *“I sort of go between [suburb 1] and [suburb 2] where I live with the youngest daughter. And then I also go up and stay [interstate], because I don't actually have a home of my own. So yeah, it's made exercise and getting into a program very difficult” (Participant L, breast cancer)* - *“busyness of Christmas and, and all the things that come with that. And like social commitments and extra work stuff. But I'm getting back into it again…I probably would have had close to a month of not exercising as much as I would normally” (Participant C, breast cancer)* | | | |
| **Guilt of knowing what to do but not doing** | - *“I probably haven't kept up as much exercise as I should have done partly because of health issues…that's my fault. That's not the program’s fault.” (Participant B, multiple myeloma)* - *“I do catch a bus up to the shops and a taxi home again once a little bit of shopping, but that just normal everyday, there's nothing extra that I really do. I know I should!” (Participant L, breast cancer)* - *“Doing the Zoom was an incentive … after six or eight weeks, that's the end of the sessions. And you're on your own. While I can do quite well, but then you literally the best will in the world, you lapse...a bit more spasmodic. I'm not saying I didn't do it. I can do it, but I'm not doing it as regularly as I would like.” (Participant N, multiple myeloma)* - *“I want to do kind of sessional program... With the exercises…On my own. Like I don't force myself to kind of do those exercises on my own” (Participant P, breast cancer)* | | | |
| **Doing what they can** | - *“but I mean, two days ago, I did the back lawn because of holidays and all that stuff. It had grown quite thick. Right, so I was out there for three and a half hours and I could do it. So that's a plus.” (Participant AC, multiple myeloma)* - *“I'm just so tired every day now that I haven't really been doing much. I go up to my shopping center for about one and a half hours and I have a bit of lunch and I walk around with it in the supermarket” (Participant J, chronic leukemia)* - *“I've just trying to do longer distances and longer. Longer [walking] sessions just for myself” (Participant P, breast cancer)* - *“I'm trying to hit 3000 steps a day, as I come out the tail end of surgery a couple of weeks ago, just trying to get back into small walks and being a bit more active than I haven't been of late” (Participant V, breast cancer)* - *“even things like as silly as it sounds, like the heel lifts… just try and get a little bit more strength and movement in my ankles, my feet. Especially since I've had the surgery, some of the arm ones to try and stretch out, as it was breast surgery” (Participant V, breast cancer)* | | | |
| **Previous exercise experience** | - *“I feel like that now that I've recovered from cancer, I wanted to get back some of my physical fitness…I used to run a bit so I thought, why not give that a shot?” (Participant I, breast cancer)* - *“I used to be pretty fit. And I and then I encountered multiple myeloma, blood cancer and the treatment of that and the effects of the disease. Slow me right down and introduce me to fatigue like I’ve have not experienced before…So I see this as a chance to look back and extend my limits and my horizons.” (Participant N, multiple myeloma)* - *“I've always been pretty active...Once I started the chemo…my strength and stamina sort of reduced a lot. So the telehealth, she really helped me to sort of stay focused” (Participant W, breast cancer)* - *“We used to walk five kilometers, every second day. My wife is in good health now. And so I'm pretty sure we'll start that again. Not so much the five km, we’ll work up to it.” (Participant AC, multiple myeloma)* | | | |
|  |  |  | |  |
